# Usefulness of ultrasonography in the emergency department to a patient with recurrent abdominal pain

**DOI:** 10.1186/2036-7902-7-S1-A8

**Published:** 2015-03-09

**Authors:** A Oviedo-García, M Algaba-Montes, J Lopez-Libano, JM Alvarez-Franco

**Affiliations:** 1 Emergency Department, Valme Hospital, Seville, Spain; 2 Critical Care Department, Miramar Hospital, Mallorca, Spain; 3 Emergency Department, IB-Salut, Ibiza, Spain

**Keywords:** Cholecystitis, Gallbladder Cancer, Free Fluid, Constitutional Symptom, Quadrant Pain

## Background

Gallbladder cancer (GC) is the most common biliary tract malignancy, representing 3% of malignant tumors, and has a high mortality, mainly related regional spread. Early detection remains difficult, and is often casual

## Objective

We present a case of GC, diagnosed at ED, through the use of US scanning used by EP

## Patients and methods

A patient with abdominal pain, with a final diagnosis of a GC

## Results

81 year old woman, was admitted to the emergency room after several consultations at its health center by right upper quadrant pain of several weeks duration, accompanied by fatigue, weight loss and a feeling of abdominal distention. On examination he had preserved the vital signs and had only found tenderness in the right upper quadrant, without signs of peritoneal irritation. Analytical emergency were unremarkable. Given the persistence of pain the EP made an ultrasound exam observing a large mass occupying the gallbladder bed, hypoechoic lesions in liver parenchyma, and perihepatic free fluid

## Conclusion

Most are adenocarcinomas (95-98%). The histological type with better survival is papillary adenocarcinoma. Produce liver metastases from expansion angiolymphatic own areas of direct hepatic infiltration. The presenting symptoms are nonspecific and difficult to differentiate from other more prevalent diseases such as biliary colic or chronic cholecystitis. The most common symptom is pain in right upper quadrant dull and aching. When symptoms such as jaundice or other constitutional symptoms usually appear advanced. US is the method of initial diagnosis image, and when it is diagnosed in early stages is usually discovered incidentally by ultrasound for another reason, which can observe a large mass occupying the gallbladder bed with wall thickening. Furthermore, US is very sensitive for detecting dilatation of intra/extrahepatic bile duct and the presence of hepatic metastatic lesions or direct infiltration of the parenchyma.

## Informed consent

The study was conducted in accordance with the ethical standards dictated by applicable law. Informed consent was obtained from each owner to enrolment in the study and to the inclusion in this article of information that could potentially lead to their identification.
